# Cross-Platform Microarray Data Normalisation for Regulatory Network Inference

**DOI:** 10.1371/journal.pone.0013822

**Published:** 2010-11-12

**Authors:** Alina Sîrbu, Heather J. Ruskin, Martin Crane

**Affiliations:** Centre for Scientific Computing and Complex Systems Modelling, Dublin City University, Dublin, Ireland; Memorial Sloan-Kettering Cancer Center, United States of America

## Abstract

**Background:**

Inferring Gene Regulatory Networks (GRNs) from time course microarray data suffers from the dimensionality problem created by the short length of available time series compared to the large number of genes in the network. To overcome this, data integration from diverse sources is mandatory. Microarray data from different sources and platforms are publicly available, but integration is not straightforward, due to platform and experimental differences.

**Methods:**

We analyse here different normalisation approaches for microarray data integration, in the context of reverse engineering of GRN quantitative models. We introduce two preprocessing approaches based on existing normalisation techniques and provide a comprehensive comparison of normalised datasets.

**Conclusions:**

Results identify a method based on a combination of *Loess* normalisation and *iterative K-means* as best for time series normalisation for this problem.

## Introduction

Identifying biological networks is an important aspect of Systems Biology, as these give insight on the complex behaviour of an organism and help find disease markers and treatments, important precepts to establish for Synthetic Biology [1]. GRNs are biological networks that describe the regulation of gene expression, fundamental to most natural processes. Computational models of GRNs enable *in silico* simulation and analysis of these processes. Quantitative models provide more information on the interactions and dynamics of the system, compared to qualitative models, but despite considerable attention in the literature [2], [3], they are still restricted in scope. This is due to the limited length of most sources of time course data, typically used for inference, which creates an under-determination problem for large GRNs.

One way of overcoming the dimensionality problem, widely recognized in the literature [4], is data integration. Inferential algorithms that integrate other types of biological measurements with microarray data have been reported [5], while integration of time series from different sources, but on the same platform, has been shown to aid GRN inference [6]. However, cross-platform integration of microarray data has been analysed only for clustering and classification problems, using normalisation techniques to remove platform and batch effects [7], [8]. An analysis of these, in the context of quantitative GRN modelling, introduces new challenges, as different pre-processing techniques may affect the data in a negative way. While most methods aim at removing noise, part of the real signal may be removed as well. This leads to over-smoothing, resulting in significant loss of information, especially when multiple consecutive normalisation stages are involved, as is the case of cross-platform normalisation. In consequence, correlations between interacting genes may be lost, or spurious correlations introduced during pre-processing, making it very difficult for inferential algorithms to uncover the real structure of the GRN. Given the nature of the data, which are highly dimensional and describe a complex system, the resulting datasets are difficult to validate, as differentiation between spurious and real correlations is hindered by complex interaction patterns. Additionally, given the quantitative nature of the models, the data used for inference need to be measuring the same quantity, whereas diverse pre-processing techniques may result in log ratios, log values or other transformed quantities, thus hindering the integration process. In consequence, two joint (single- and dual-channel) pre-processing approaches based on existing normalisation techniques are introduced here, to reconcile these quantities, and a comparison framework is built for assessment of results.

## Methods

### Data

Normalisation analysis has been performed on four distinct, publicly available, raw datasets, representing microarray time series measurements during the Yeast *Saccharomyces Cerevisiae* cell cycle. These include three dual-channel, (Spellman [9], PramilaS [10], PramilaL [11]), and one single-channel dataset, (Hasse [12]). Each of these analyses two cell cycles, at different time intervals. The Spellman dataset contains 18 time points sampled every seven minutes, measured using c-DNA microarrays, while the PramilaS dataset contains 13 time points, sampled every 10 minutes on *Amplicon v1.1* microarrays,(c-DNA). The PramilaL dataset contains 25 time points, sampled every 5 minutes on the same Amplicon platform, and features a dye-swap replicate, which is used in our experiments as a second time series of the same length. The Hasse dataset contains 15 time points, sampled with *Affymetrix* arrays every 16 minutes, and a replicate that is again used as a separate time series during inference. This results in six time series measurements of the cell cycle, sampled at different intervals. The common genes in these datasets were extracted, resulting in 5337 genes for analysis.

### Normalisation techniques

The normalisation performed in this study consists of two stages. Initially, noise pre-processing was performed, using three different approaches. On the resulting datasets, three cross-platform normalisation techniques were applied, resulting in a total of nine normalised datasets for comparison. Additionally, the time spans were scaled so that the cell cycle length is the same across datasets.

The first pre-processing stage aims at noise reduction within each dataset, to prepare it for cross-platform normalisation. Several normalisation techniques exist in the literature [13], especially tailored for single- and dual-channel arrays, where a channel represents a different sample [13]. However, these methods usually yield data of different type and scale, i.e. log ratios for dual-channel and ‘absolute’ expression values for single-channel, which are difficult to integrate in a qualitative model. In this context, three different approaches, (one standard and two integrative), were used for within-dataset normalisation and compared for each dataset previously described.

The first approach, *PMLoess*, applies different normalisation techniques depending on platform type: PMOnly, (available in the dChip software, [14]) for Affymetrix, and Loess normalisation, (available in the Limma Bioconductor package, [15]), for dual-channel data. PMOnly was chosen as a preferred method in previous studies [16], while Loess normalisation is an established method for pre-processing dual-channel arrays [13]. The logarithm of expression levels resulting from dChip was computed for the Affymetrix dataset, to obtain semantics similar to log ratios obtained after Loess normalisation for the dual-channel datasets.

Additional normalisation aims at reconciling use of both log ratios and log values by applying Loess normalisation to Affymetrix data and PMOnly normalisation to dual-channel data. These two methods are, henceforth, refered to as *LoessOnly* and *PMOnly*. *LoessOnly* applies Loess normalisation [15] to both dual- and single-channel arrays, by considering the average of the perfect-match probes to be the red channel, and the mismatch probes to be the green channel, where red and green correspond to the two samples compared in dual channel arrays. In dual-channel datasets (PramilaL, PramilaS and Spellman), the red channel corresponds to samples taken at the different time points during the cell cycle, and the green channel to a control sample, which is the same for all time points. In single-channel data, both perfect-match and mismatch probes correspond to the same sample, where the sample values are different at each time point. However, given that mismatch probes measure unspecific hybridisation, (genes can hybridise even if their sequence is not the correct complement of the probe), and that the amount of sample solution used in each experiment is the same, the mismatch signals should be close to one another at different time points. Thus, correspondence applies between the green channel in dual-channel time series and the mismatch probes in single-channel series. *PMOnly*, on the other hand, applies dChip to both types of data, taking the background-normalised red channel to be a perfect match probe.

For each dataset resulting from the first pre-processing stage, we applied cross-platform normalisation techniques, as follows. (i) A simple standardisation on each dataset, 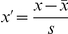, for data values 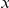 with sample mean 
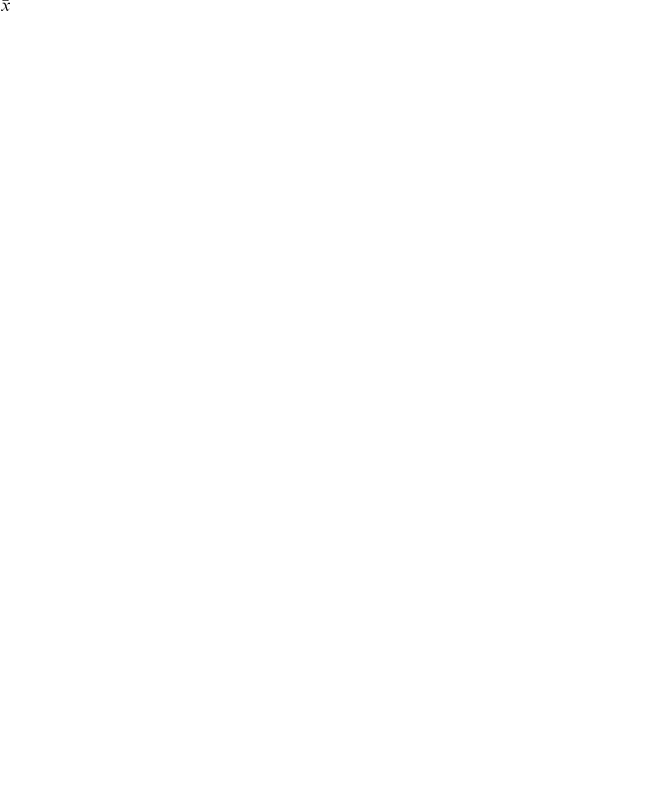
 and sample standard deviation 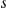 was performed [8]. This was followed by a scaling of values to lie on the interval [0,1], which restricts the data to the same range. The scaling was performed by subtracting, from all values, the minimum expression level over all four datasets (plus a predefined 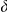), followed by dividing all values by the maximum (plus 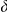). The restriction to interval (0,1) was necessary as the model used here (described in Section *Evaluation criteria*) requires positive expression values for all genes, and facilitates computation by restricting its output to the same interval. (ii) ComBat [7], a Bayesian technique aimed at removing batch effects, and (iii) XPN [8], a cross-platform normalisation technique based on iterative K-means clustering, were also applied for cross-platform normalisation (outlines of the two methods are included in the following paragraphs). Additionally, scaling onto the interval [0,1], was performed, as noted. All these techniques aim at standardising data *across platforms*, after a preliminary normalisation within each dataset. The implementations, made available by the authors, were used for the latter two methods. The final datasets are identified in this paper by the name of the normalisation techniques used for each stage: *PMLoess* methods (*PMLoess_St*, *PMLoess_ComBat*, *PMLoess_XPN*), *PMOnly* methods (*PM_St*, *PM_ComBat*, *PM_XPN*) and *LoessOnly* methods (*Loess_St*, *Loess_ComBat*, *Loess_XPN*). The rest of this section briefly describes the cross-platform normalisation procedures ComBat and XPN.

**ComBat** [7] is a normalisation method for eliminating batch effects, which models the gene expression level for gene 
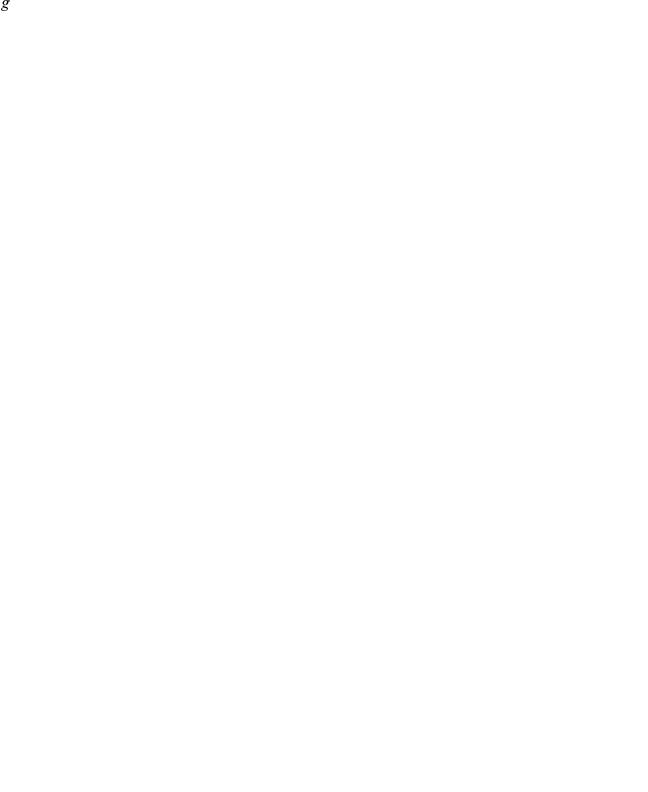
 in experiment 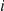 and platform 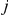 as:
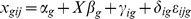
(1)with 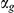 the overall expression level, 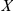 a design matrix for experiment conditions, 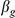 the vector of regression coefficients for X, 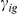 and 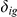 the batch effects, and 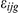 the noise term (normally distributed with zero mean and 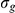 variance).

The method consists of three steps. (i) The data is standardised to obtain similar overall mean and variance for genes. This involves fitting of parameters 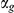, 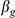 and 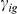 by using a least-squares approach, estimation of 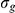, and computation of a standardised data point as:
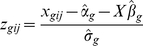
(2)Further (ii), the batch effect parameters are estimated, using the assumptions that 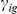 are normally distributed (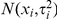) while 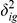 follow the 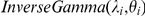 distribution. The parameters for these distributions are estimated using the method of moments. Finally (iii), the data are adjusted for batch effects:
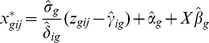
(3)A more detailed description of this normalisation approach can be found in [7].

**XPN** [8] is a cross-platform normalisation procedure based on the assumption that subsets of genes have the same pattern in subsets of experiments. The expression level for a gene 
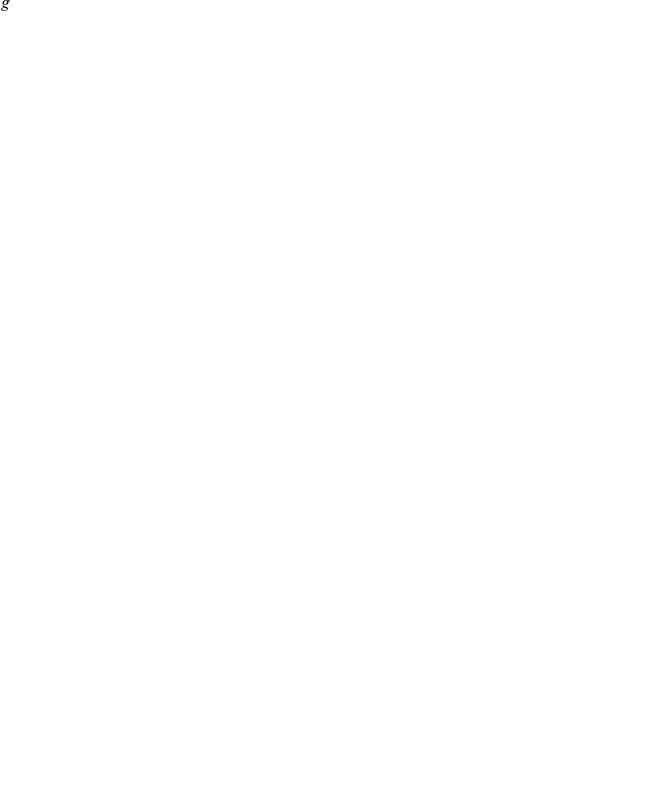
 in sample 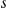 and platform 
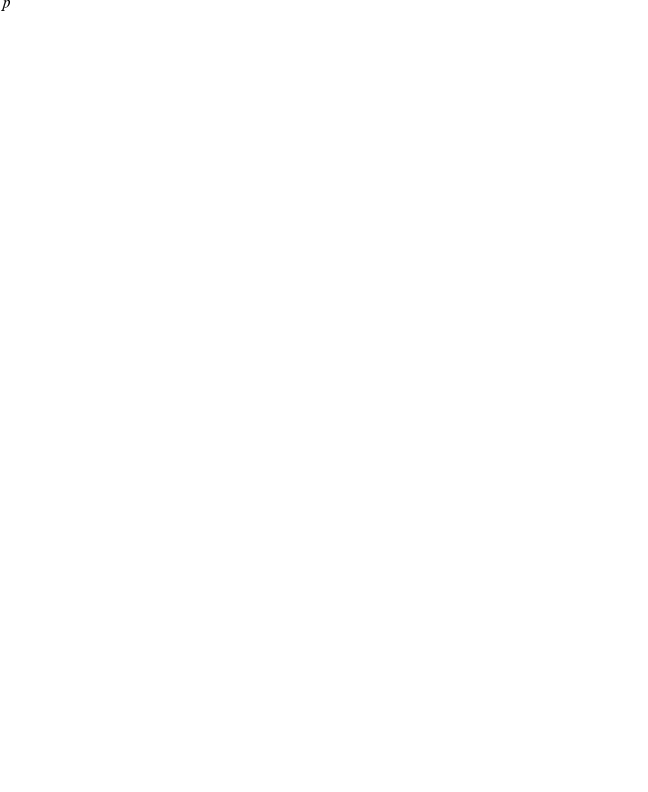
 is considered to be a *block mean*, 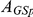, which is the same for a subset of samples (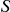) and genes (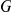), and common across platforms (
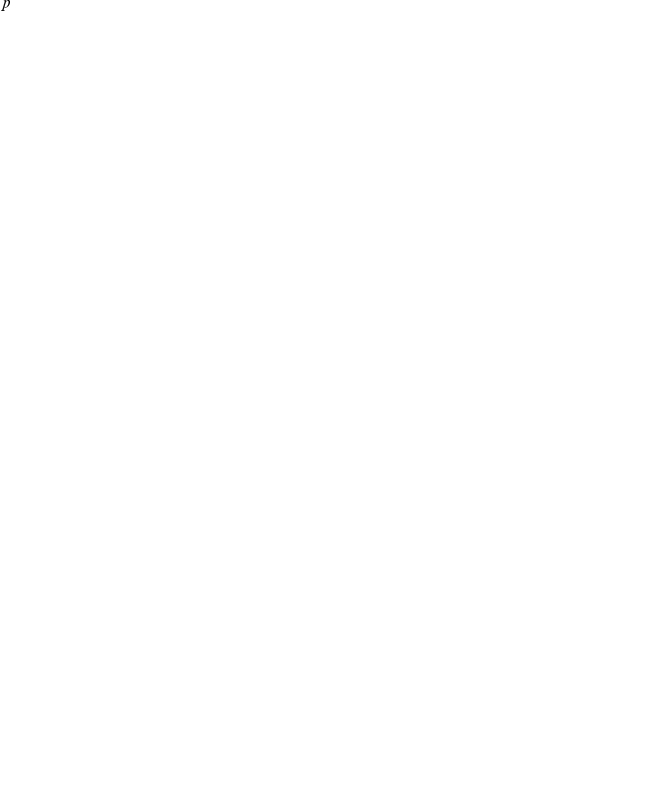
), transformed by a scaling and a shifting factor, i.e. 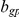 and 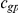, specific to each gene (
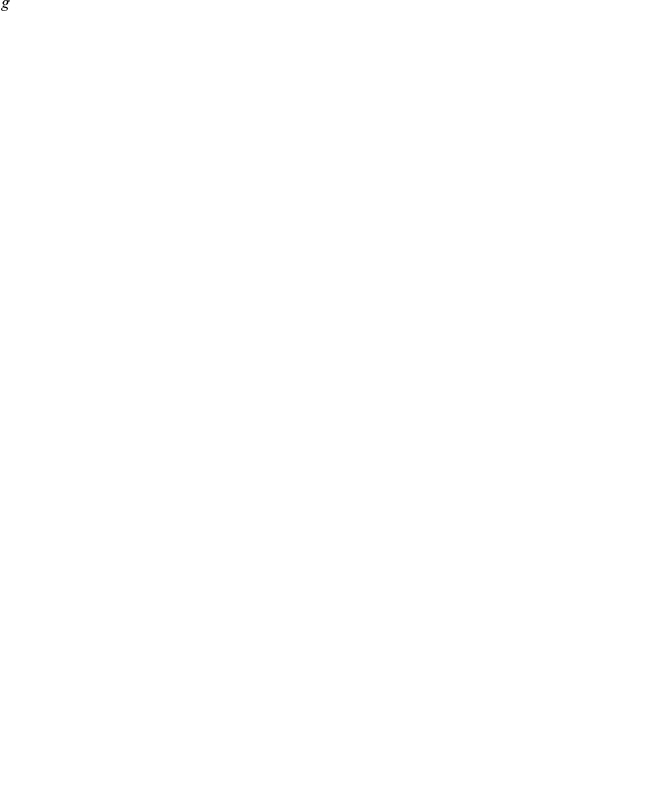
) and platform (
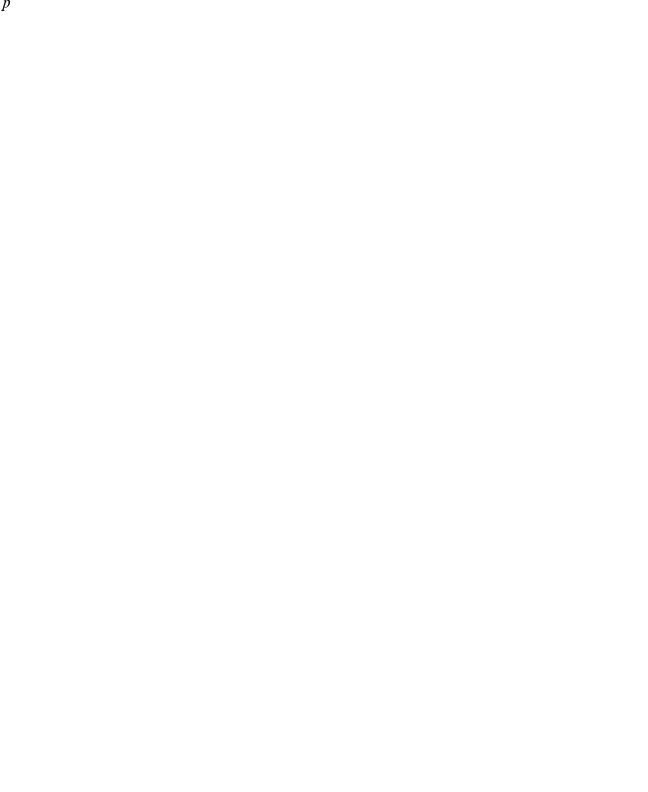
), and a noise term 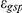, (specific to each gene, sample and platform):
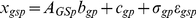
(4)In order to find 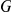 and 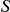, i.e. the groups of genes and samples where the block mean values apply, K-means clustering is applied separately on sample and gene patterns obtained by combining the datasets to be normalised. Based on cluster assignment, the model described in Equation 4 is fitted to the data, using a maximum likelihood method. Normalised expression values are computed based on the model obtained:
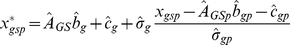
(5)where 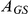, 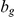, 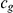 and 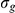 are weighted averages of parameters 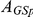, 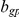, 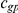 and 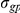, obtained for each platform. The procedure is iterated 30 times to obtain 30 normalised values, corresponding to different cluster assignments, and final expression values are computed as the average of the values obtained in each run. More details on this normalisation procedure can be found in the original paper, [8].

### Evaluation criteria

Evaluation of the normalisation methods applied has been carried out using four different criteria. Firstly, (i) variability between replicates has been computed, as the average over all genes of the rMSE (squared root of Mean Squared Error) between replicate expression values, normalised by the average gene expression level for each gene, (Equation 6).
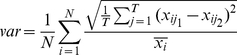
(6)Here, 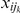 represents the expression level of gene 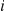 in experiment 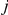 and replicate 
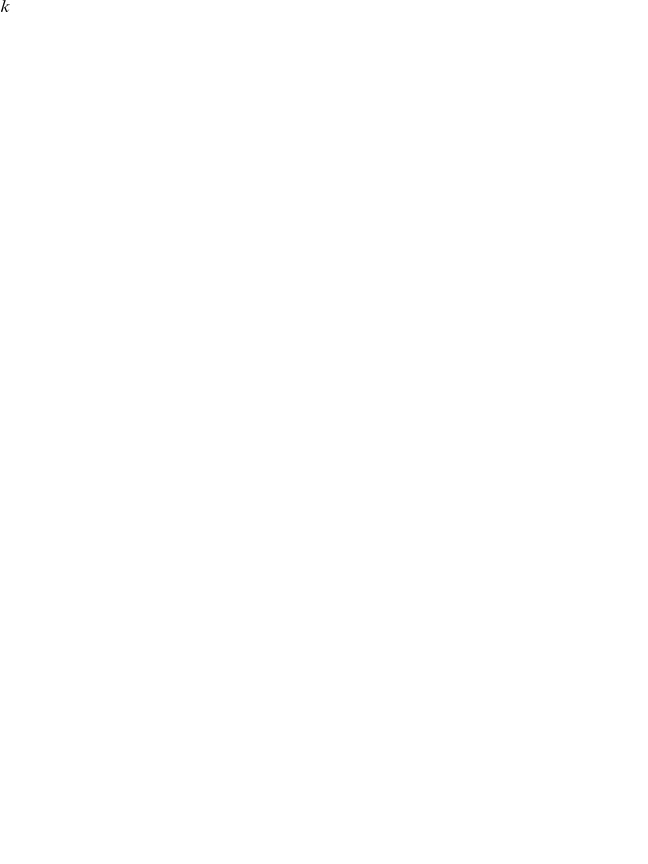
, 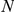 is the total number of genes and 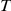 is the total number of experiments. The datasets used contain a dye-swap replicate for one dual-channel dataset (PramilaL), (where the same two samples are hybridised in two consecutive experiments, but the dyes used for each sample are swapped) and one technical replicate for the single-channel (Hasse) dataset (the same sample is used in two consecutive experiments). This allows for a comparison on both replicate types. Ideally, after normalisation, replicates should be approximately the same, so the distance between them is a criterion widely used for validation of normalisation techniques, e.g. [16].

Secondly, (ii) wavelet analysis was used to compare the normalisation techniques used. Wavelets [17] are a mathematical tool for time-scale signal analysis: at large scale, low frequencies present in the signal can be readily extracted, while small scale analysis detects high frequency components. In signal decomposition in general, high frequencies correspond to noise, while low frequencies correspond to the signal itself [17]; this also applies to time series gene expression measurements. Here, we have used discrete wavelet decomposition to obtain wavelet coefficients for gene signals at different scales, (also known as *levels*). This type of decomposition uses a set of functions, called wavelets, which are generated by contracting and dilating a base function, i.e. the *mother wavelet*, in discrete steps [17]:
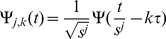
(7)Here, 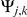 is the wavelet obtained from 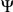, the mother wavelet, by using 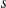, a fixed scaling step, which is usually 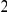, and 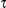, a translation factor, usually 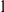. This results in a discrete sampling of the time-scale space. The resulting wavelets are used to represent the signal as a discrete superposition:
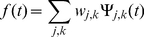
(8)where 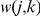 represent the wavelet transform coefficients, which describe components of the signal, corresponding to scale window 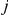 and time window 
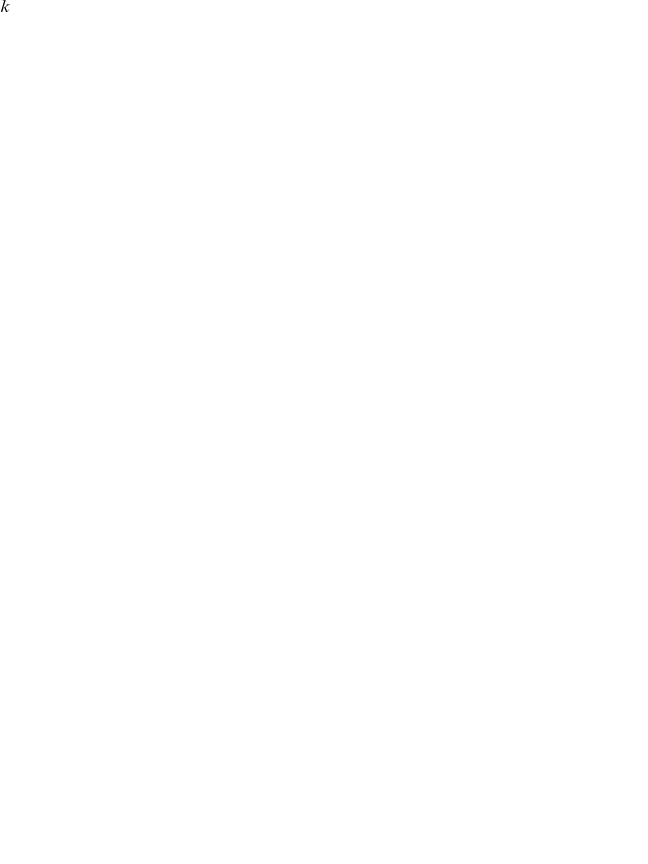
. In practice, to obtain these coefficients, an iterative approach is used, which builds coefficients for the upper half of the frequency spectrum (considering 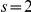 and 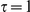), filters these frequencies out and repeats the process for the lower half, after sub-sampling the signal by 2. This results in different *levels* for coefficients, with low levels corresponding to small scale i.e. high frequencies, and high levels to high scale i.e. low frequencies. Having 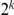 time points in the data, 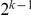 level 1 coefficients are computed, for short time windows (total time divided by number of coefficients), 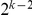 level 2, for double-sized time windows, while the last level, 
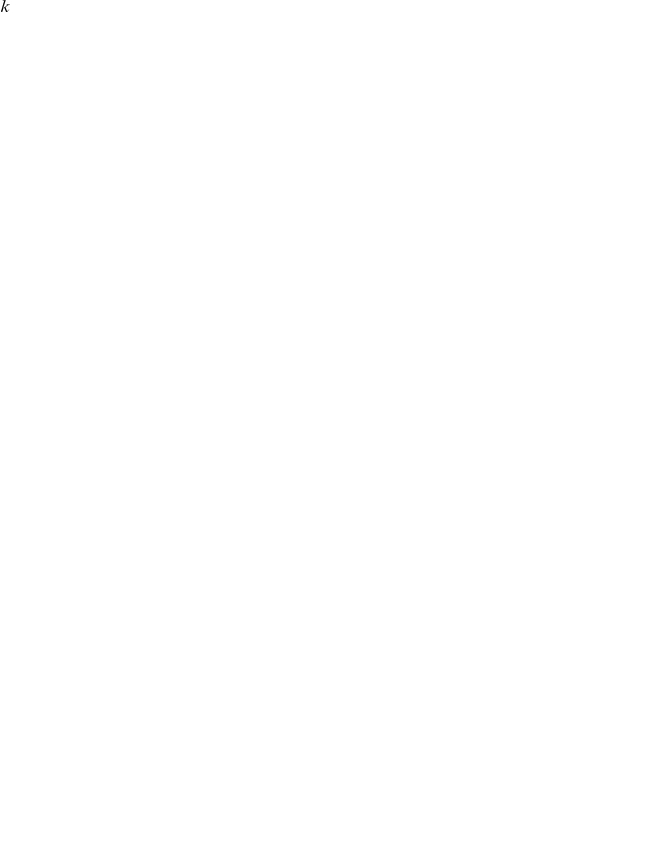
, contains just 2 coefficients, for large time windows. Each of these coefficients indicates the amplitude of the current frequency spectrum present in the signal in the current time window. This results in high time resolution and low frequency resolution at small scale and high frequency resolution and low time resolution at large scale.

Here, we have used Daubechies mother wavelets for analysis [17]. The signals corresponding to gene expression time series were re-sampled to obtain 32 time points and, after decomposition, 32 wavelet coefficients on 5 levels (scales). Level 1 coefficients describe the amplitude of the highest frequencies in the signal, while level 5 corresponds to the lowest frequencies. The average absolute value of the high frequency coefficients, corresponding to 9 genes known to be involved in the cell cycle, (from Kegg database [18]), was computed, as these components are a good indication of the magnitude of noise in the data. Also, wavelet coefficients for gene signals from different datasets were compared at different scales (by computing RSS -Residual Sum of Squares- values), in order to assess which normalisation techniques bring the data closer together. All computations were performed using the Matlab toolbox WaveLab [19].

Thirdly, (iii) a correlation analysis was performed to test whether correlations vary between normalisation techniques, as well as to determine whether genes known to interact are correlated after normalisation. This is important for GRN model inference, as interacting genes will have correlated expression levels across time [20]. Normalisation may remove useful correlations, along with noise, or may introduce spurious correlations in the data [21]. The Pearson correlation coefficient [22] was computed between all gene pairs and, given the high dimensionality of the obtained correlation matrix, three aggregation criteria were used for analysis. Additionally, correlations between known interacting genes were calculated.

The first aggregated criterion used was the number of gene pairs with absolute correlation larger than 0.9, which was compared across normalised datasets, to determine how each normalisation technique affects high correlations. Secondly, the average of absolute correlations for each gene 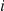 was computed as shown in Equation 9.
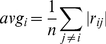
(9)where 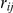 represents the Pearson coefficient between genes 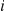 and 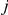 while 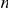 is the number of gene pairs. These values give a measure of how the gene relates to the rest of the system. Thirdly, the correlation variability between microarray datasets (Spellman, Hasse, PramilaL, PramilaS) *within* each normalised dataset was computed, for each gene pair. Ideally, the same pair of genes should have similar correlation across microarray datasets, but, due to platform differences and normalisation, these can vary. The correlations common to the different datasets are most reliable, while others are more likely to be spurious, i.e. introduced by the platform or normalisation technique, (although some differences may appear due to the removal of useful correlations by the normalisation process in part of the datasets). In this work, the correlation differences between microarray datasets was computed as indicated in Equation 10:
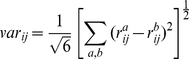
(10)where 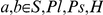 and 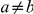, 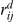 represents the Pearson coefficient between genes 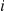 and 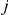 in dataset 
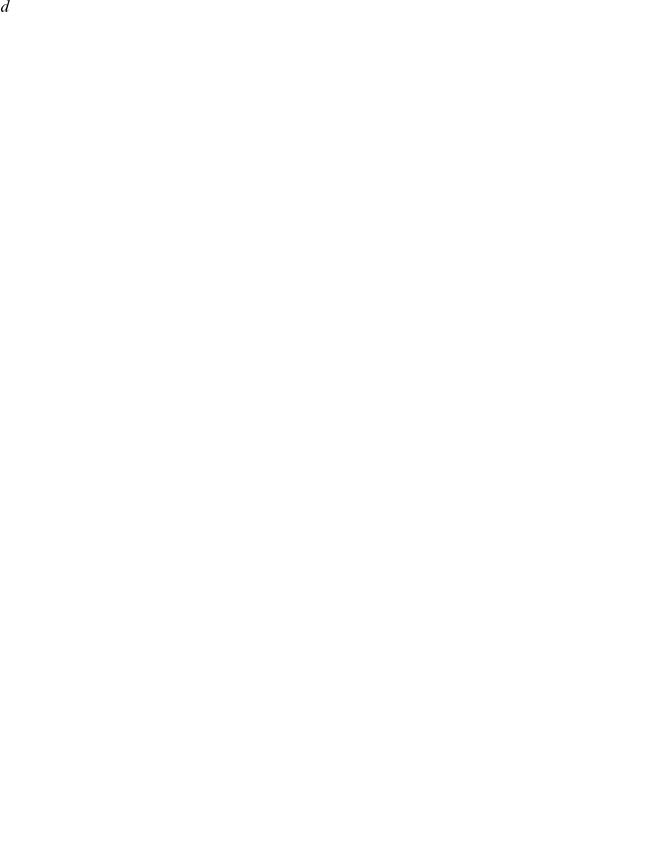
, with 
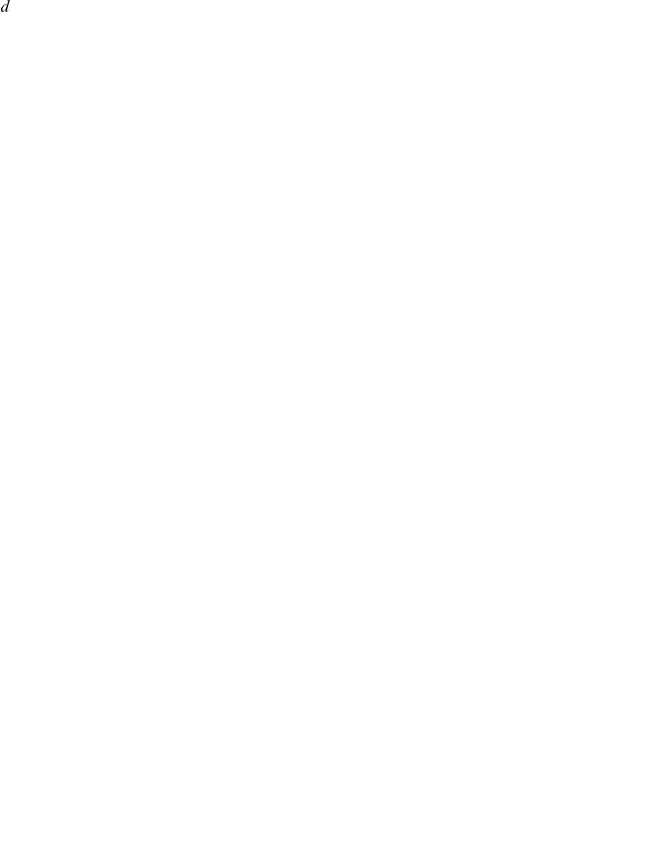
 having values 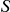 (Spellman), 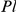 (PramilaL), 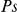 (PramilaS) and 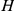 (Hasse). This results in a matrix, for each normalisation technique, referred to as *correlation variability matrix* in the rest of this paper, which shows how correlations between pairs of genes differ from one microarray dataset to another. This can be viewed as an indicator of the amount of spurious correlation in each normalised dataset. Here, we use the *average* of the values in the correlation variability matrix to quantify this, because of similar high dimensionality of these matrices.

A different method of identifying spurious correlations would be to analyse partial correlation coefficients in the data, i.e. the correlation seen after removing effects from other genes, as opposed to zero-order coefficients such as Pearson, which consider the correlation in isolation. However, this is difficult to assess here, as the pattern of covariance is very complex, with many gene pairs having high zero-order correlation and known existence of circuits in the causality networks, hence our use of the correlation variability matrix, described above, as a (weaker) criterion.

The fourth evaluation criterion used was (iv) the capability of single gene models to translate between datasets. For this, models were built from each dataset individually, and then were applied to simulate the same genes in the other three datasets. An inferential algorithm, based on evolutionary computation, [23], was used to build S-System models of regulation for two genes (CLN1, CLN2) in a 9-gene network, (chosen from the Kegg database to be loosely connected to the rest of the cell cycle GRN). S-Systems [24] are systems of differential equations based on the power-law formalism, and have been previously used for GRN modelling [3], [25]:
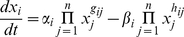
(11)Here, 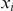 denotes the expression level of gene 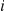; thus, the first and second terms represent the synthesis and degradation of mRNA, which are influenced in a positive or negative way by the genes in the network. The rate constants, 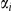 and 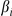, represent basal synthesis and degradation rate, respectively, while 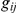 and 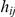, the kinetic orders, indicate the strength of influence of gene 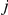 on synthesis and degradation of gene 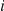, respectively. Positive values of 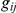 indicate activation of gene 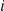 by gene 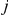, while negative values indicate repression. For the purpose of this paper, the decoupled version is used, where model parameters for each gene are inferred separately [23], as opposed to determining parameter values for the whole system at once. Even though outcomes may be influenced by the inferential technique, the error between these simulations and the real signal seen in the test datasets is still a very good indication of how close the datasets are and, consequently, of how the normalisation technique performs. Due to the stochastic nature of evolutionary algorithms, 20 runs were performed for each inference task, and rMSE values, normalised by the mean expression values (rMse/Mean), were averaged across these.

Additionally, models have been inferred from combining two datasets, and testing on a third, to analyse how the data fit changes compared to using each training dataset individually. This demonstrates that data integration can improve inference, and enables analysis of the effect of each normalisation technique. The same error measure, i.e. rMSE/Mean, has been used to evaluate the difference between simulated and experimental data.

## Results and Discussion

### Variability analysis

**Figure 1:**
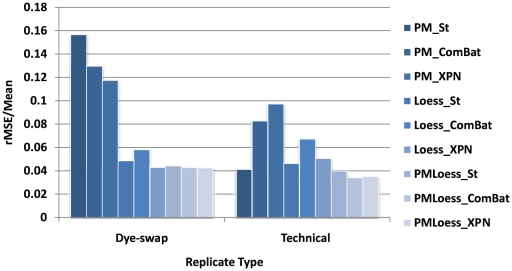
Variability between replicates in 9 datasets obtained by different normalisation techniques. The graphs show average rMSE/mean (Equation 6) values for dye-swap (dual-channel arrays, PramilaL dataset) and technical replicates (single-channel arrays, Hasse dataset).

Figure 1 indicates that PMOnly methods display increased variability in both dye-swap (dual-channel) and technical replicates (single-channel). LoessOnly methods, (established technique for dual-channel arrays), exhibit low fluctuation even in single-channel technical replicates, indicating that, although not developed for this type of data originally, they perform well with respect to the variability criterion. Also, ComBat and XPN give increased variability between replicates compared to standardisation in some cases, showing that cross-platform normalisation comes with a cost in terms of replicates.

### Wavelet analysis

**Figure 2:**
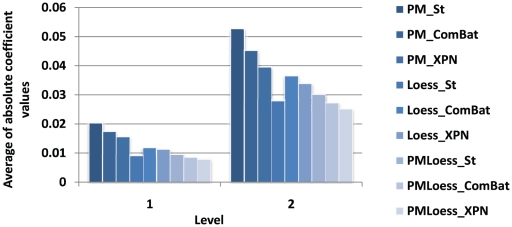
Magnitude of high frequencies. Graph shows average absolute value of wavelet coefficients for levels 1 and 2, corresponding to highest frequencies in the data, i.e. noise. Averages are computed over all four datasets.

A first analysis, based on wavelet decomposition, measures the amplitude of high frequencies in the different normalised datasets. High amplitudes indicate stronger noise compared to low amplitudes. Figure 2 shows average absolute values for wavelet coefficients for the highest frequencies in the data, over all four datasets. Results show that PMOnly methods display the largest fluctuations, while PMLoess methods give the smallest. This was expected to some extent, as the latter methods apply normalisation techniques especially tailored for each type of data. Again, LoessOnly methods display good behaviour, very close to PMLoess. However, in LoessOnly, ComBat and XPN seem to increase variability, in contrast to PMOnly and PMLoess, where variability decreases. This is in agreement with the replicate variability analysis (Section *Variability analysis*), and shows, again, that cross platform normalisation may come with a cost from the variability viewpoint.

**Figure 3:**
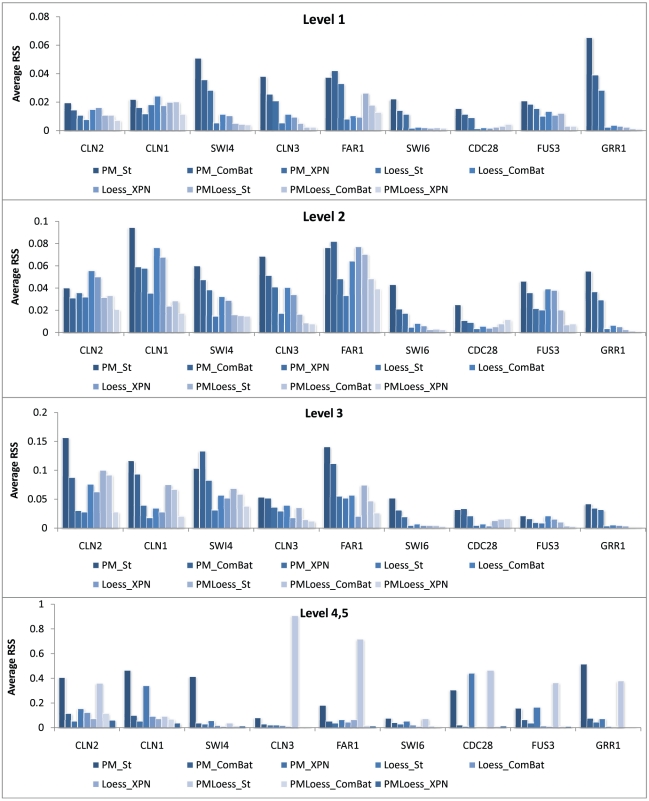
Dissimilarity between gene signals in different datasets. Graphs show average RSS between wavelet coefficients corresponding to nine genes in the four datasets, at different scales(levels). Level 1 corresponds to highest frequencies, i.e. noise, while level 4 and 5 to lowest frequencies, i.e. the real signal.

A second application of wavelet decomposition, for assessment of pre-processing methods, compares coefficients, at different scales, for signals describing expression levels for the same gene occurring in different datasets. Here, nine genes known to be involved in the cell cycle, (analysed as a GRN also in Section *Model translation*), are compared across the four datasets, and results are summarised in Figure 3. This shows that for levels 1,2 and 3, (corresponding to higher frequencies), PMOnly methods show the largest differences between gene signals, for most genes analysed, while LoessOnly and PMLoess are comparable. This is probably due to the high variability in PMOnly data (when analysing high frequencies only), noted earlier in this section. However, more relevant is the behaviour seen for levels 4 and 5, which contain coefficients that describe the real signal, as these differences indicate how different the core gene expression levels are. As Figure 3 shows, cross-platform normalisation methods bring the data significantly closer together, compared to simple standardisation. The behaviour at levels 4 and 5 is also reflected at previous levels, although differences are smaller.

### Correlation analysis

**Figure 4:**
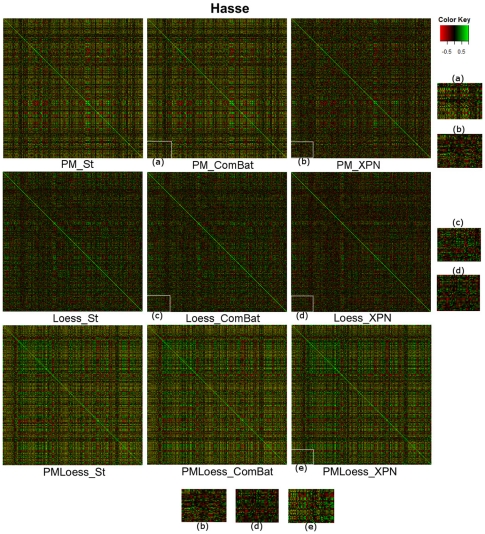
Correlation matrices for Hasse dataset (single-channel), for each normalisation technique. Enlarged areas, labelled (a), (b), (c), (d), (e) are provided for better visualisation. Images show that XPN cross-platform normalisation decreases high correlations compared to ComBat and Standardisation ((a) vs. (b) and (c) vs. (d)) while Standardisation and ComBat yield same correlation values. Also, PM methods display a higher number of strong correlations ((a) vs. (d) and (e)). These effects are studied further in text by providing values for aggregated criteria from the correlation matrices.

**Figure 5:**
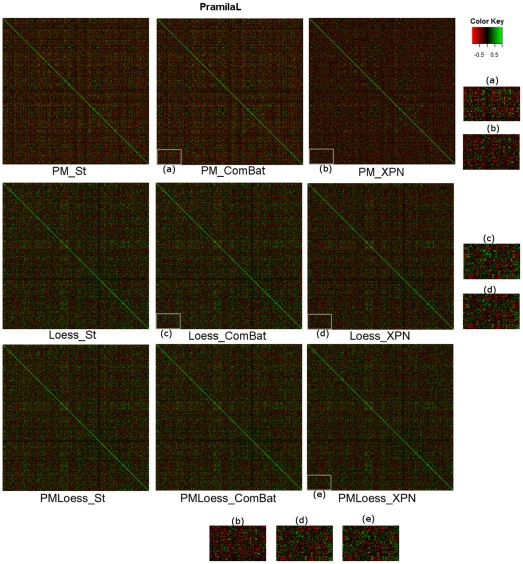
Correlation matrices for PramilaL dataset (dual-channel), for each normalisation technique. Enlarged areas, labelled (a), (b), (c), (d), (e) are provided for better visualisation. Behaviour similar to that for the Hasse dataset can be observed, with decreased amount of high correlation for XPN cross-platform normalisation, and with high correlations for PMOnly methods. However, differences are smaller than for the Hasse dataset, indicating that single-channel data is more sensitive to the normalisation approach taken.

In order to provide an overall view of the correlation distribution in the normalised datasets, Figures 4 and 5 display *heatmaps* of the pairwise correlation matrices, for datasets Hasse (single-channel) and PramilaL (dual-channel). These indicate that Loess and XPN methods decrease pair-wise correlations compared to PM and Standardisation approaches, with much larger differences seen in the single-channel (Hasse) dataset. Due to the large dimensionality of these heatmaps, two aggregated criteria have been used for further analysis. Table 1 summarises overall values obtained for different cross- and within-platform normalisation, including the variability criteria used in previous sections, to support the discussion on correlations.

**Table 1:** Summary of variability and aggregated correlation values for different within- and cross-platform normalisation.

	Cross-platform						Within-platform					
	Standardi-sation		ComBat		XPN		PMOnly		Loess Only		PMLoess	
	SC	DC	SC	DC	SC	DC	SC	DC	SC	DC	SC	DC
Variability between replicates	↑ with PML; ↓ with PM & L	↑ with PM & PML; ↓ with L	↑ with PM & L; ↓ with PML	↑ with L; ↓ with PM & PML	↑ with PM & L; ↓ with PML	↓	↑	↑	↓	↓	↓	↓
Amplitude of noise frequencies	↑ with PM & PML; ↓ with L		↑ with L; ↓ with PM & PML		↑ with L; ↓ with PM & PML		↑		↓		↓	
Number of highly correlated genes	↑	↑	↑	↑	↓	↓	↑	↑ in PL & PS; ↓ in S	↓	↓	↑	↑ in S; ↓ in PS & PL
Average absolute correlation	↑	↑	↑	↑	↓	↓	↑	↓	↓	↑	↑	↑

SC and DC identify results for single- and dual-channel datasets; PL, PS and S represent the three dual-channel datasets (PramilaL, PramilaS and Spellman), while PM, PML, L stand for PMOnly, PMLoess and LoessOnly, respectively. Arrows indicate whether variability and correlations are increased (↑) or decreased (↓) relative to the other normalisation procedures in the same category (cross- or within-platform).

**Figure 6:**
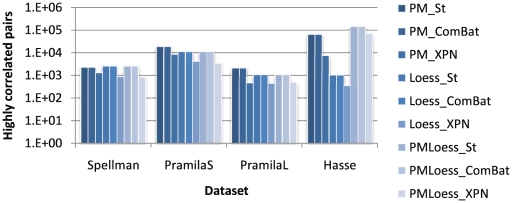
Number of highly correlated gene pairs in each dataset, for each normalisation technique (in logarithmic scale). The correlation threshold used was 0.9.

Firstly, the number of highly correlated gene pairs has been studied in each dataset. The correlation threshold used was 0.9, and Figure 6 shows the number of gene pairs with absolute correlation larger than this, for each normalised dataset. Results show a very large difference on the log scale between normalisation techniques used. PMOnly methods display a large number of highly correlated gene pairs in the Hasse dataset and in two of the three dual-channel datasets (PramilaS and PramilaL), while LoessOnly methods eliminate a large part of these correlations, especially in the Hasse dataset. The question here is whether this high number of correlations is an artefact of the PMOnly normalisation method, or whether Loess methods do, in fact, substantially decrease correlations. A second important observation is that ComBat and Standardisation display the same correlation values, while, in comparison, XPN causes significant decrease in the number of high correlations for all datasets.

**Figure 7:**
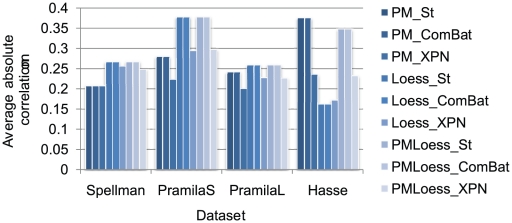
Average correlation for gene SWI4. This shows an aggregated measure of correlation of this gene with all other genes in the network, for each normalisation technique. Note that ComBat cross-platform normalisation does not affect correlations, while XPN decreases the average values.

Secondly, the average of absolute correlations for a subset of nine genes was computed, with results for gene SWI4, (which is a known transcription factor involved in cell cycle regulation), shown in Figure 7. For the dual-channel datasets, (Spellman, PramilaL, PramilaS), LoessOnly methods show large average correlation, in contrast to the low number of highly correlated pairs, noted for the same methods (previous paragraph). This suggests that, for dual-channel data, large correlations are only slightly decreased by LoessOnly methods, whereas, (given the large variability for PMOnly), the larger number of highly correlated genes may be an artefact of the PMOnly normalisation technique. For the single-channel dataset Hasse), however, the average correlation is decreased by Loess normalisation, and, considering the significant drop in highly correlated gene pairs, it can be concluded that, although PMOnly normalisation may lead to spurious correlations in the Hasse dataset, Loess normalisation may also decrease correlations for these data, so a further analysis for quantifying spurious correlation has been performed.

#### Correlation variability matrix

**Figure 8:**
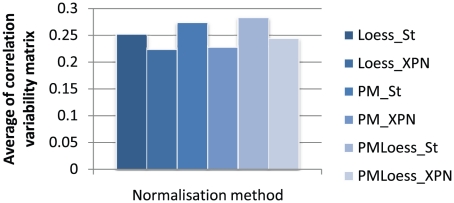
Average of correlation variability matrix. The correlation variability matrix measures correlation differences between datasets, as defined in Equation 10. Plotted here is the average of values in matrices for different normalisation procedures. Note that LoessOnly methods display lowest differences, indicating less presence of spurious correlation, and better agreement between datasets. XPN normalisation also decreases differences, compared to standardisation. Thus Loess_XPN exhibits fewest differences, closely followed by PM_XPN.

In order to assess the amount of spurious correlation for each normalisation technique, the *correlation variability matrix* (Equation 10) was computed for each normalisation procedure, and averages over all gene pairs (i.e. all elements in these matrices), are shown in Figure 8. ComBat does not affect correlations, compared to standardisation for cross-platform normalisation, so the corresponding datasets, (i.e. PM_ComBat, Loess_ComBat and PMLoess_ComBat), are not included in the analysis. Results show that Loess methods display smaller averages compared to PM, while XPN is lower still, indicating less spurious correlation. In conclusion, Loess_XPN exhibits the best behaviour, from the variable correlation point of view, as coefficients are in good agreement across microarray datasets. This performance is closely followed by that of PM_XPN. This similar behaviour indicates that the effect of cross-platform normalisation on the correlation differences is larger than the effect of within dataset normalisation, which is to be expected. Given the use of the correlation variability matrix as a criterion for analysing spurious correlation, it can be argued that agreement between datasets may be due just to systematic bias in the normalisation procedure. Although this can not be ruled out, correlation agreement is still required for data integration, so it may be concluded that methods that display large correlation variability perform less well. For a further study of quality of correlation, a small number of genes known to interact are analysed in the rest of this section.

#### Analysis of genes known to interact

**Figure 9:**
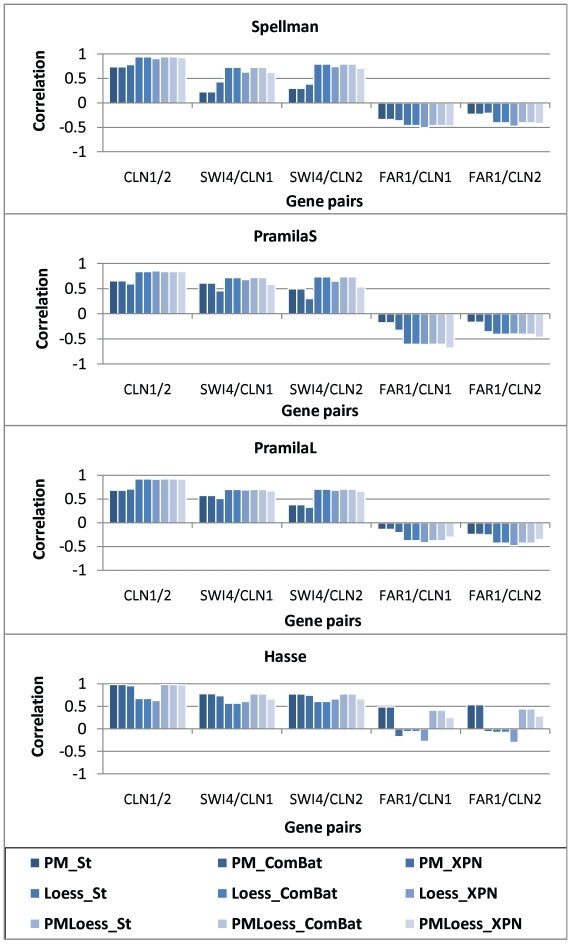
Correlation between genes known to interact. The first three pairs of gene are positively interacting, and the positive correlation values correctly indicate the interaction type, in all datasets. The fourth and fifth gene pairs, on the other hand, should display negative correlations, as they are repressor/target pairs. However, while for the dual-channel datasets this relationship is confirmed by negative correlations, in the Hasse dataset it is only visible with PM_XPN and LoessOnly methods, with Loess_XPN displaying largest absolute value. This indicates that Loess_XPN enhances correlations in this case.

In the context of GRN modelling, it is very important that interactions between genes correspond to correlations seen in the data. To analyse this, we have chosen a set of 5 gene pairs, which are known to interact in reality ([18]). These include pairs (a) CLN1/2 of genes working together as a complex, (i.e. co-regulated), (b) SWI4/CLN1 and (c) SWI4/CLN2, where SWI4, in a protein complex, is known to activate genes CLN1/2, and the pairs (d) FAR1/CLN1 and (e) FAR1/CLN2, where FAR1 represses the formation of CLN1/2. Ideally, for (a), (b), (c), a high positive correlation should be seen in the data, while for (d) and (e), a high negative correlation should be present. Figure 9 shows correlations for each dataset, and each normalisation technique.

For the first three datasets, (Spellman, PramilaL, Pramilas - dual-channel), Loess normalisation displays better behaviour, with PM-only methods giving significant decrease in correlations between genes known to interact. It is important to note that the correlation values do correctly indicate the nature of these interactions, with positive values for (a), (b), (c), and negative for (d) and (e). However, correlations between CLN1/2 are higher than those corresponding to activation/repression pairs, which can be explained by the regulatory time delay, which represents the time elapsed between the expression of the regulator and that of the regulated gene, causing a shift in the expression signal of the target, compared to the regulator, and, consequently, decreasing correlation values between the corresponding time series. For the Hasse dataset, on the other hand, the negative correlation between FAR1/CLN1/2 is not present, except after Loess normalisation, and, even then, absolute values are very small. This supports the hypothesis that PMOnly methods introduce spurious correlations into the data, probably due to the high variability, (discussed in Sections *Variability analysis* and *Wavelet analysis*). For the other gene pairs, positive correlations are decreased using Loess, (Hasse dataset), but agreement with values obtained for dual-channel datasets (i.e. PramilaL, PramilaS and Spellman) remains good.

It is very important to note, when analysing gene pairs known to interact, that, although overall average correlations are smaller, as noted earlier, XPN does not decrease correlations in all cases; some increases are observed, compared to other methods. This, combined with the low correlation variability shown previously, indicates that XPN reduces spurious high correlations, as opposed to ‘useful’ ones, which it conserves or even amplifies, even in datasets such as Hasse, (Figure 9), where other techniques fail to do so.

### Model translation

**Figure 10:**
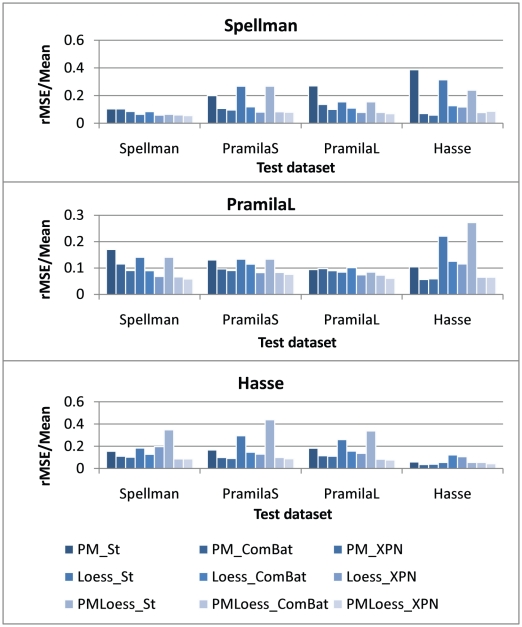
Average rMSE/Mean on all datasets for 20 S-System models for gene CLN2. Models were inferred from datasets Spellman, PramilaL and Hasse, separately, (identified by graph titles), and then tested on the rest of the datasets (horizontal axis). Graphs show that cross-platform normalisation, other than standardisation, decreases fitting errors for the test datasets.

Applying models, built from one dataset, to others, can indicate whether pre-processing improves agreement between datasets, i.e. which genes are involved and co-regulated in the measured process. To assess this, we have computed the average rMSE/Mean between simulations of 20 S-System models for each dataset and the real expression values. Models were obtained though evolutionary optimisation, which is a stochastic process, so multiple runs were performed for a robust analysis of results. rMSE/Mean values are displayed in Figure 10, for gene CLN2 models inferred from Spellman, PramilaL and Hasse datasets. These show that, in general, cross-platform normalisation, (as opposed to simple standardisation), significantly decreases error on all test datasets, making it a very important step in data integration for GRN modelling. Also, it is important to note that PMOnly and LoessOnly methods display behaviour comparable to *combined PMLoess* methods, indicating that these normalisation approaches are also suitable for time series model inference. Similar results were obtained for gene CLN1, but are not shown here.

#### Combining datasets

**Figure 11:**
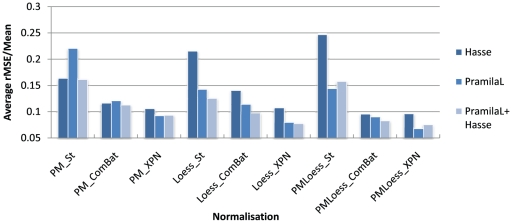
Average rMSE/Mean on the Spellman dataset for gene CLN2. Twenty inference runs have been performed with datasets Hasse, PramilaL and Hasse+PramilaL combined, and average errors, tested on the Spellman dataset, displayed for each normalisation technique. These show that, for PMOnly and LoessOnly methods, behaviour on the test dataset improves when using combined data, regardless of the cross-platform normalisation technique used, while for PMLoess methods this happens only for ComBat cross-platform normalisation. This is a good indication that these within dataset normalisation methods improve integrated data inference. Loess_XPN displays lowest rMSE values, suggesting that this is a suitable normalisation method for cross-platform data integration.

In order to test how data integration improves model inference with different normalisation techniques, a second analysis was performed. This involved inferring models for the same gene (CLN2) from datasets PramilaL and Hasse together and testing these on the Spellman dataset. The resulting error, (averaged over 20 runs), has been compared to that obtained by models inferred from PramilaL and Hasse individually, with results displayed in Figure 11, as rMSE/Mean values. This shows that, for most normalisation techniques, increasing the number of datasets incorporated in model inference decreases the error when this model is subsequently applied to the test dataset. The exceptions are PMLoess_St and PMLoess_XPN, where the error for the models inferred, from the PramilaL dataset alone, is smaller than that found when the Hasse data are also included in the training set. This is not too surprising since, in these cases, within dataset normalisation is different for dual-channel (PramilaL and Spellman) and single-channel (Hasse) data, resulting in log-ratios for the former and log-values for the latter. Consequently, model performance, tested on the Spellman dataset, is decreased by including the Hasse dataset in the training set. In PMLoess_ComBat, the increase in error when using two datasets is not visible, even though this method also uses different within dataset normalisation for single- and dual-channel data. This may indicate that the cross-platform normalisation employed (i.e. ComBat) is better able to eliminate platform differences, in this case. The decrease in error on the test dataset for PMOnly and LoessOnly methods, when using two training datasets as opposed to one only, indicates that the integrative within dataset normalisation procedures introduced here (PMOnly and LoessOnly) do aid combined data inference, by reconciling different quantities, resulting from typical Loess and PMOnly normalisation.

Based on lowest error obtained for model inference, (using two datasets as opposed to one), Loess_XPN performs best, providing strong indication of its suitability as a normalisation method for data integration in GRN modelling.

### Conclusions

Three pre-processing approaches (LoessOnly, PMOnly and LoessPM) have been applied to integrated raw microarray data from 3 different platforms. This has included application of techniques developed for dual-channel, (Loess [15]), on single-channel data and vice-versa, (PMOnly [14]). Following initial within-sample pre-processing, three cross-platform normalisation techniques, (Standardisation, ComBat [7] and XPN [8]), were applied, resulting in 9 normalised datasets. These have been compared for four criteria, relevant for data integration in the context of GRN quantitative modelling: variability between replicates, wavelet coefficient analysis, simple gene-gene correlations and GRN differential equation model translation between datasets.

From the variability viewpoint, LoessOnly methods performed better than PMOnly, although combined PMLoess methods exhibited best performance overall. Wavelet analysis and model translation indicated that a second normalisation stage, (cross-platform), as opposed to simple standardisation, is required in order to align the datasets for the same inferential process. However, variance is increased for experimental replicates by cross-platform processing. Additionally, combining datasets was shown to increase performance on a test dataset, especially when using integrated-within dataset normalisation, with best data fit obtained by Loess_XPN. Analysis of correlation between genes showed that Loess methods decrease high values, although patterns between genes that are known to interact are preserved. XPN also reduces some highly correlated gene values, but, in many cases, correlations between genes known to interact are amplified, even for those gene pairs for which other methods failed to obtain the correct correlation sign. This suggests that it is a fairly sensitive probe for determining true interaction patterns in the data.

In conclusion, results indicate that Loess_XPN was found to be best for normalisation of time-series data for quantitative model inference, as variability is acceptably low, datasets are well aligned, correlations between interacting genes are enhanced and models, obtained from combined datasets, perform better on test data than models inferred from one dataset only. The method permits integrated pre-processing across platforms, facilitating model inference from heterogeneous datasets.
